# Novel Bacteria-Immobilized Cellulose Acetate/Poly(ethylene oxide) Nanofibrous Membrane for Wastewater Treatment

**DOI:** 10.1038/s41598-019-55265-w

**Published:** 2019-12-12

**Authors:** Doaa Zamel, Ahmed H. Hassanin, Rania Ellethy, Gamal Singer, Ahmed Abdelmoneim

**Affiliations:** 1 Biochemistry Department, Faculty of Science, Helwan University, Helwan, Egypt; 2 Material Science and Engineering Department, Egypt-Japan University of Science and Technology, New Borg El Arab, Alexandria, 21934 Egypt; 3 Textile Engineering Department, Faculty of Engineering, Alexandria University, Alexandria, Egypt; 4 Nanoscience Program, Institute of Basic and Applied Sciences, Egypt-Japan University of Science and Technology, New Borg El Arab, Alexandria, 21934 Egypt

**Keywords:** Biological techniques, Biotechnology, Chemical biology, Ecology, Microbiology, Environmental sciences, Chemistry, Energy science and technology, Materials science, Nanoscience and technology

## Abstract

In this study, electrospun cellulose acetate - poly(ethylene oxide) nanofibrous membrane was found to be unique in immobilizing bacterial cells. Here, removal of methylene blue in aqueous media was achieved by using isolated species of bacteria (*Bacillus paramycoides*) from industrial wastewater and immobilized on cellulose acetate- poly(ethylene oxide) nanofibers using DMSO as a solvent. The decolorization time was varied from 0 to 72 h, different dye concentrations from 20 to 200 mg/L and bacterial cells count was investigated to achieve the maximum MB removal by bacteria-immobilized CA/PEO nanofibrous membrane. The effective dye decolorization was achieved within 48 h and MB removal % was around 93%. Furthermore, reusability of the bacteria-immobilized CA/PEO nanofibrous membrane was tested. It was found that after the 4^th^ usage, 44% of the dye decolorization capacity still could be achieved. These results are promising and suggest that bacteria-immobilized CA/PEO nanofibrous membrane could be economically feasible and eco-friendly when used in MB removal from industrial wastewater. Combination of both adsorption and biodegradation methods was found to be effective in MB removal from aqueous media.

## Introduction

Dyes are used in industry in order to give color to several products such as textiles, leather, plastics and paper^1^. Many dyes are produced with huge amounts around 280,000 tons per year^2^. Most dyes do not bind to the target material, and this subsequently results in 10–50% excess dye that was discharged directly into wastewater^3^. Likewise, approximately 5000 tons of dyes are exhausted into effluents annually^4^. As a result, their discharge into the environment is a matter of concern as they may cause pollution for both toxicological and esthetical scales^5^. Methylene blue dye is a thiazine cationic dye which has widespread applications in industry, likewise in dying cotton, silk and wool^6–8^. However, it may cause eye burns which lead to permanent eye injuries in human and animals^9,10^. Besides, it may give rise to short periods of rapid heartbeats or difficult breathing in case of inhalation. Indeed, treatment of any effluent containing MB dye is of great importance due to its harmful impacts on water quality and perception. Different treatment methods were performed for effluents discharged from industries to decolorize dyes; such as biodegradation^6,10^, chemical oxidation^11^, foam flotation^6^, electro-coagulation^7^, adsorption^10,12,13^ and photodegradation by Titanium Dioxide^13,14^. However, the efficient, eco-friendly and cost-effective method for MB removal from aqueous systems remains a challenge. The biodegradation and biosorption methods of dyes using microorganisms such as bacteria, fungi and algae have been extensively cited, as they are considered as cost-effective and eco-friendly methods for dye removal^3,15^. Compared to other microorganisms, bacteria can decolorize a wide range of dyes with high efficiency as they are easier to culture, have a rapid growth and the capability to degrade pollutants under a wide range of environmental harsh conditions. Bacteria do not consume MB dye for nutrition, instead they perform biodegradation as a defense mechanism against the dye toxicity. The use of free bacterial cells in methylene blue biodegradation from industrial wastewaters has been formerly reported^3,16^. However, free bacterial cells in general cannot be harvested from wastewater after application, hence this may develop another unavoidable source of pollution, and consequently they could not be applied on the industrial scale. The use of bio-integrated support for immobilizing bacteria could solve those drawbacks and bring additional advantages over free bacterial cells usage such as lower space and growth medium necessities, potential reusability and higher resistance to environmental extremes^17^. Due to simplicity, versatility and cost-effectiveness of electrospinning technique, besides its ability to control fiber morphology (e.g. higher surface area and porosity), nanofibrous membranes have been recently presented as promising support for immobilization of microorganisms used in dye bioremediation and water purification applications^18,19^. Previous studies on cellulose acetate electrospinning has been showed that it is a versatile material for fabrication as it is water-insoluble, biodegradable, biocompatible and highly porous good support for bacteria immobilization^16,20–23^. Furthermore, cellulosic nanofibers exhibit a very high surface area which enriches their adhesion properties and this could be useful in bacterial immobilization^24^. Yet, several studies in the literature on immobilization of microorganisms on nanofibrous membranes suggest that the generated systems have good potential for use in many environmental practices. In a study of San *et al*.^25^, showed the effect of cellulose acetate (CA) nanofibers on immobilization of bacteria in MB decolorization by surface attachment method. Another study, *Acinetobacter calcoaceticus* STB1 cells were immobilized on electrospun CA nanofiberous mats in order to achieve enhanced ammonium removal in aqueous environments^26^. However, the weak adhesion properties between bacteria and nanofibrous membranes represent major obstacles in the real life application of these newly generated systems on the industrial scale. Likewise, bacteria shall be fallen easily from the nanofibers when applied in water systems. Hence, encapsulation of bacteria inside nanofibers using electrospinning technique could be a pathway for efficient trapping of bacteria inside nanofibers and it recently appeared in the literature. For instance, *Escherichia coli* and *Staphylococcus albus* were embedded in poly(vinyl alcohol) solution and water was the solvent, the results showed the potential of electrospinning process in immobilization of both bacterial strains on PVA nanofibers^27^. Furthermore, the encapsulation of *Lysinibacillus* sp. bacteria in water- soluble and biocompatible non-polymeric cyclodextrin fibers (CD-F) using electrospinning process has been recently touched before^28^. However, PVA and CD are water soluble and could not be effectively applied in wastewater applications. From that approach, cellulose acetate was best chosen as it exhibits water in-soluble properties. Nevertheless there are previous studies for CA electrospinning using organic solvents such as acetic acid, acetone and dimethyl fluoride, all these solvents are toxic on bacteria which may lead to bacterial death. Consequently, the choice of an organic solvent for CA electrospinning that is safe on bacterial cells remains a challenge. This is why it was arranged to prepare cellulose acetate/poly(ethylene oxide) nanofibrous membrane using dimethyl sulfoxide (DMSO) as a new solvent because it is safe on bacterial cells and maintains their viability^29–31^. Furthermore, the present work aims to investigate the electrospinning technique on the immobilization of the isolated *Bacillus paramycoides* on cellulose acetate- poly(ethylene oxide) nanofibers and asses the combination of the biodegradation and adsorption methods on methylene blue removal. This novel membrane could be easily handled, affordable, economical and reusable for more than three times in industry.

## Results

### Morphological characterization of bacteria-free CA/PEO and bacteria-immobilized CA/PEO nanofibers

**Figure 1:**
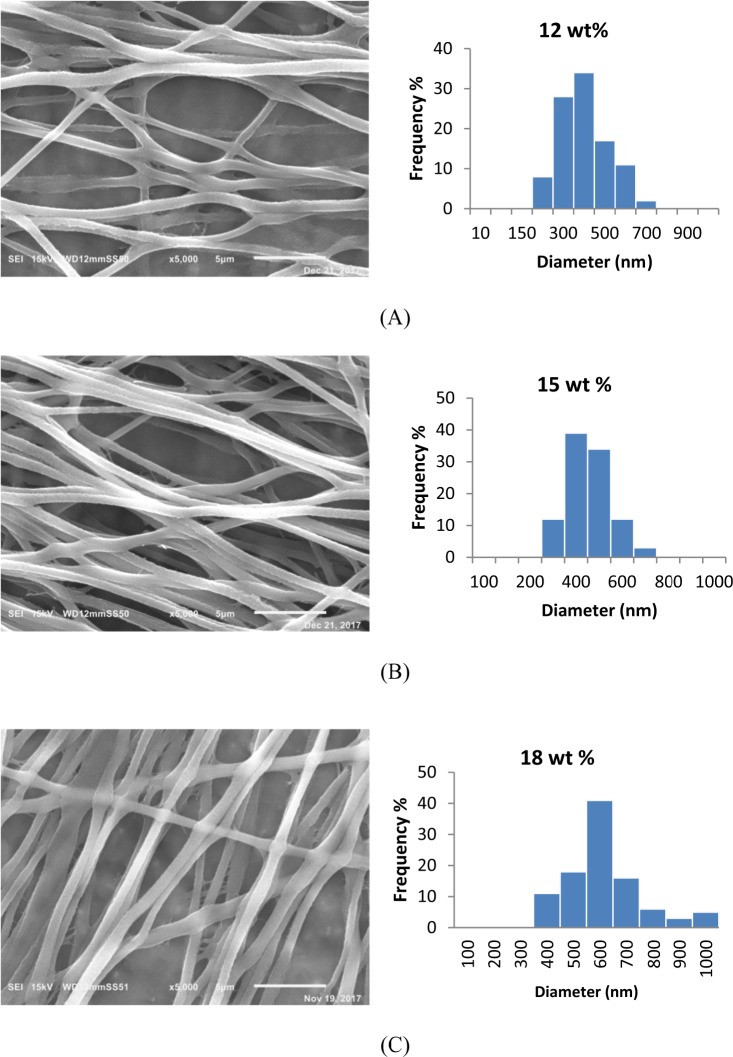
Representative SEM images of cellulose acetate- poly(ethylene oxide) nanofibers at different concentrations and fiber diameter distribution (**A**) 12; (**B**) 15 and (**C**) 18 wt%.

**Figure 2:**
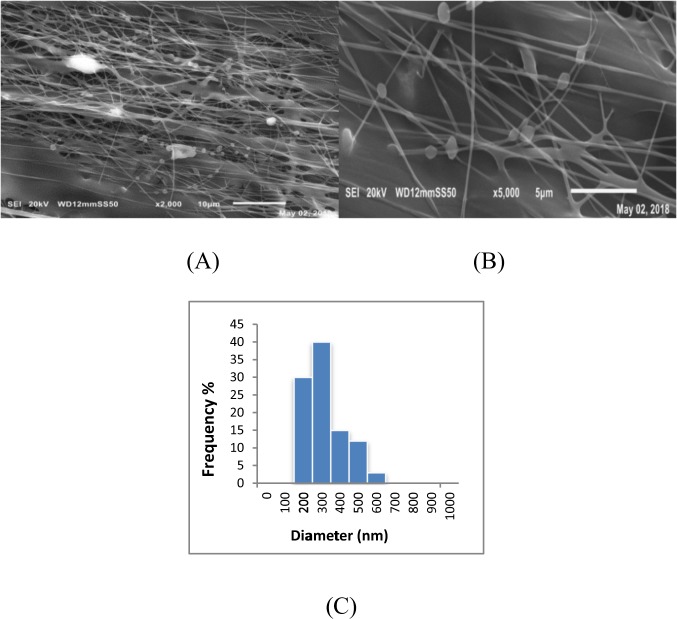
SEM images of Bacteria-immobilized CA/PEO nanofibrous membrane and fiber diameter distribution; (**A**) at low magnification, (**B**) at high magnification and (**C**) fiber diameter distribution.

The morphologies of cellulose acetate- poly(ethylene oxide) and Bacteria-immobilized cellulose acetate- poly(ethylene oxide) nanofibers were investigated using scanning electron microscope (SEM) as shown in Fig. 1. As there is a lack in research studying DMSO as a solvent for cellulose acetate in electrospinning and nanofibers production, optimum conditions for the electrospinning such as polymer concentration had to be optimized. Generally, bacteria-free CA/PEO solution was successfully electrospun into bead-free and uniform fibers that have smooth morphology and the nanofibers prepared from solution precursor containing 15 wt% CA/PEO showed the least mean average in fiber diameter distribution. Therefore, it was selected as an ideal matrix for trapping bacterial cells and examining their distribution and morphology after electrospinning. The incorporation of *Bacillus Paramycoides* into CA/PEO blend solution did not significantly affect the electrospinning and nanofibers were successfully produced (Fig. 2). In a closer look, bacterial cells were successfully encapsulated within the CA/PEO fiber matrix, forming local widening in the fiber. Interestingly, the *Bacillus Paramycoides* cells changed its typical rode shape to nearly round shape once upon subjected to the electrospinning process. This change in shape of bacteria cells could be due to the effect of electrostatic field generated during the electrospinning process by the application of high voltage (30 KV).

### Methylene blue (MB) decolorization results

To determine the maximum MB dye removal by the bacteria-immobilized CA/PEO membrane, the effects of contact time, initial dye concentrations, and bacteria-immobilized CA/PEO membrane area on the decolorization of MB were investigated (Table 1).

**Table 1:** Design of experiment for the investigated parameters.

Factors	Levels
Contact time, h.	5 (4, 12, 24, 48, 72)
Dye concentration, mg/L	4 (20, 50, 100, 200)
Membrane area, cm^2^	3 (1, 2, 4)

#### Effect of contact time

**Figure 3:**
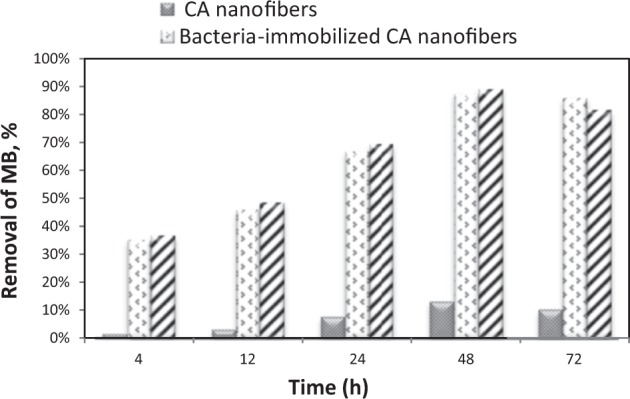
Comparison between MB removal % achieved by CA/PEO nanofibers, bacteria-immobilized CA/PEO nanofibrous membrane and free bacteria at different time intervals.

Contact time is one of the important parameters to achieve the effective dye removal in practical applications. The optimum removal time of MB by bacteria-immobilized CA/PEO nanofibrous membrane was determined. The MB decolorization performances of free bacteria, CA/PEO nanofibrous membrane and bacteria-immobilized CA/PEO nanofibrous membrane after different time intervals (4, 12, 24, 48 and 72 h) were tested. From Fig. 3, the maximum MB removal % achieved by CA/PEO nanofibers was 13.3% after 48 h. This can be attributed to the high surface area and the pores in CA/PEO nanofibrous membrane which lead to dye adsorption on the surface of CA/PEO nanofibers. On the other hand, MB removal % by free bacteria and bacteria-immobilized CA/PEO nanofibrous membrane were increased dramatically to reach the maximum level after 48 h, 89.13 and 87.39% respectively. After that, the MB removal % declined after 72 h to be 81.74 and 85.91% in free bacteria and bacteria-immobilized CA/PEO nanofibrous membrane, respectively.

#### Effect of dye concentration

**Figure 4:**
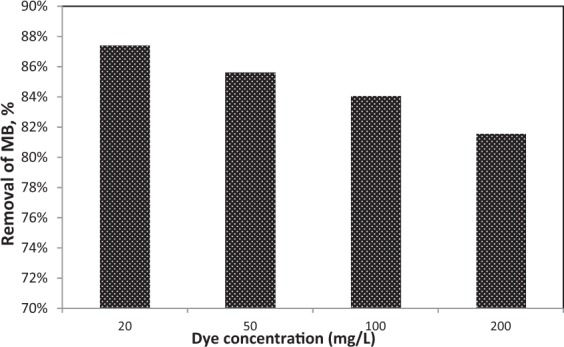
The effect of different dye concentrations on the decolorization yield after 48 h incubation period.

From literature, the effluent of MB dye ranging from 10–200 mg/L into industrial wastewater, gives it a deep blue color^25^. Therefore, different dye concentrations were studied ranging as 20, 50, 100 and 200 mg/L. Constant pieces of nanofibers were put onto different flasks containing different dye concentrations. Bacteria-immobilized CA/PEO nanofibrous membrane has shown efficient MB removal % within 48 h. In addition, effect of dye concentration on removal % using bacteria-immobilized CA/PEO nanofibrous membrane was investigated at different initial dye concentrations ranging 20–200 mg/L at pH 7 (Fig. 4). At the end of the 48 h incubation period, the maximum removal % of 20 mg/L MB solution by bacteria-immobilized CA/PEO nanofibrous membrane was 87.39%. However, the removal % decreased with an increase in the dye concentration. For 50 mg/L dye concentration, the MB removal % was 85.62%. When dye concentration is increased to 100 mg/L, the decolorization capacity was 84.06%. In the case of 200 mg/L MB concentration, the MB removal % was 81.56%.

### Effect of increasing bacteria/CA/PEO membrane

**Figure 5:**
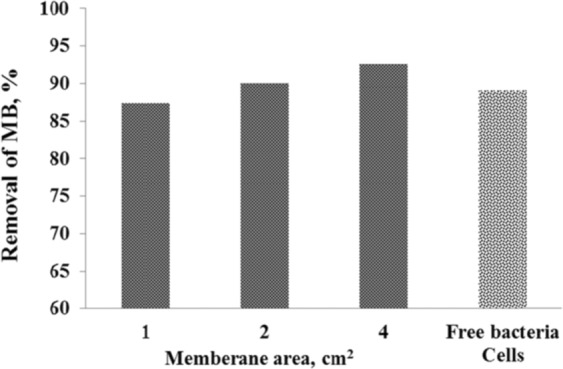
Effect of increasing bacterial cell count and membrane area on MB removal % after the 48 h incubation period.

As illustrated in Fig. 5, effect of increasing the bacteria-immobilized CA/PEO nanofibrous membrane was investigated using constant dye concentration of 20 mg/L and 48 h contact time. In the prepared bacteria-immobilized CA/PEO nanofibrous membrane samples, the piece of 1 cm^2^ of the nanofibrous membrane contains 4.23 × 10^8^ bacterial cells. Therefore, 2 cm^2^ contains 8.46 × 10^8^ and 4 cm^2^ contains 16.92 × 10^8^ bacterial cells. Likewise, the bacterial cells count was increased with increasing the pieces area of the nanofibrous membrane as 1, 2 and 4 cm^2^. As a result, the bacterial cells count and the nanofibrous membrane area have shown an obvious effect on MB dye decolorization.

### Langmuir adsorption isotherm

**Figure 6:**
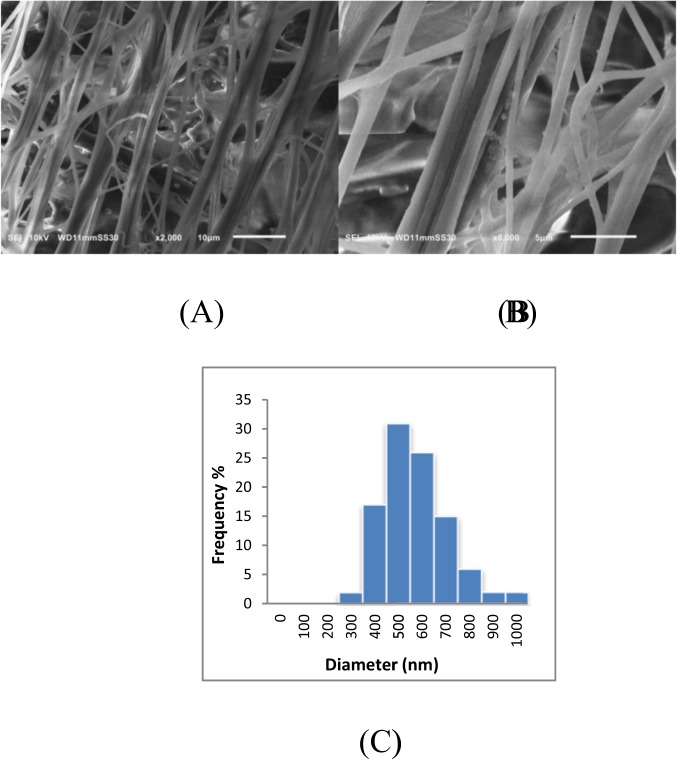
SEM images of bacteria-immobilized CA/PEO nanofibers after dye removal and fiber diameter distribution; (**A**) at low magnification, (**B**) at high magnification and (**C**) fiber diameter distribution.

Langmuir adsorption isotherm has the special significance of being the model that applies to ideal case of physical or chemisorption on a smooth surface with no interactions between adsorbed molecules. It is shown in Fig. 6, Langmuir model fits correctly for modeling the adsorption of MB on CA/PEO nanofibers. The statistic determination coefficient (R^2^) is very close to 1 which indicates regular Langmuir isotherm model. Furthermore, it implies the behavior of the adsorption is monolayer. The simplest theoretical equation representing adsorption isotherms that characterize the dependence of MB removal function “θ“ on the time is that due to Langmuir which is given by Eq. 1^30^.

1 $$\frac{{\rm{\theta }}}{1-{\rm{\theta }}}={\rm{KC}}$$

In which θ could be obtained from relationship 2:

2 $${\rm{\theta }}=1-\,\frac{{{\rm{C}}}_{{\rm{i}}}}{{{\rm{C}}}_{{\rm{o}}}}$$

Where (C_i_) and (C_o_) are the residual and initial concentrations of MB respectively. In addition, K is the equilibrium constant of the adsorption reaction which is related to the sorption energy, it is the affinity of adsorbent towards the adsorbate. High values of K imply to a strong binding and low values indicate a weak binding. Whereas, C is the adsorption time given in hours.

Free energy could be calculated from Eq. 3:

3 $$\varDelta {\rm{G}}^\circ =-\,{\rm{RT}}\,\mathrm{ln}\,{\rm{K}}$$

Where:

R: the universal gas constant (8.314 J. mol^−1^ K^−1^), T: temperature (K), and K: the equilibrium constant.

From Langmuir linear model, K = 0.0033

Thereby, ∆G° = 14.62 KJ.mol^−1^ ^34^

### Fiber morphology after MB decolorization experiments

**Figure 7:**
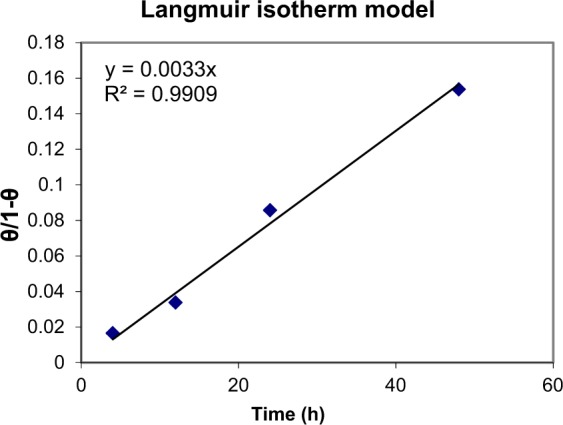
Fitting of Langmuir model to the adsorption results of MB by CA/PEO nanofibers.

To follow up the change in the nanofibers morphology after 48 h incubation period, SEM characterization was performed on the fiber membrane after 48 h incubation period. As seen in Fig. 7, no significant damage in the fiber morphology is observed and fibers maintain their smooth appearance, except the swelling of fibers due to water absorption by the fibers. It is further shown that, MB dye diffused between the fibrous matrix and adsorped on the fiber surface.

### Reusability experiments for bacteria-immobilized CA/PEO nanofibrous membrane

**Figure 8:**
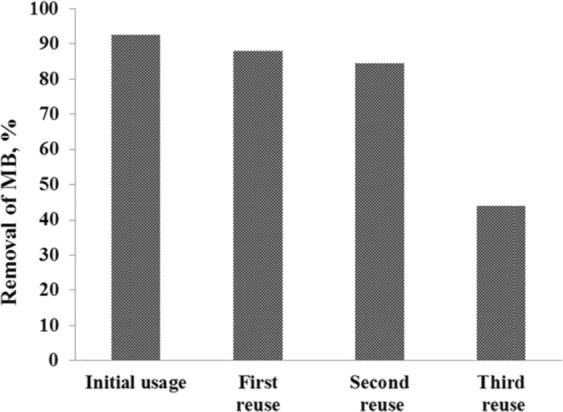
Reusability test results of the four usages on MB removal %.

Reusability was studied on the resulted bacteria-immobilized CA/PEO nanofibrous membrane for three times after the initial usage by washing with distilled water before each usage. Reusability tests were studied after 48 h incubation period, 20 mg/L MB, and 35 °C incubation temperatures. From Fig. 8, it was observed that the MB removal % decreased by increasing the reusability usage number. Approximately 44% of the dye removal % was obtained after the final usage (4^th^ usage) which suggests that the bacteria-immobilized CA/PEO nanofibrous membrane could sustain their biodegradation capacity after several times of usages and may be reused repeatedly for dye removal from wastewater in textiles and paints industry which maximize its economic feasibility for industrial applications.

## Discussion

**Figure 9:**
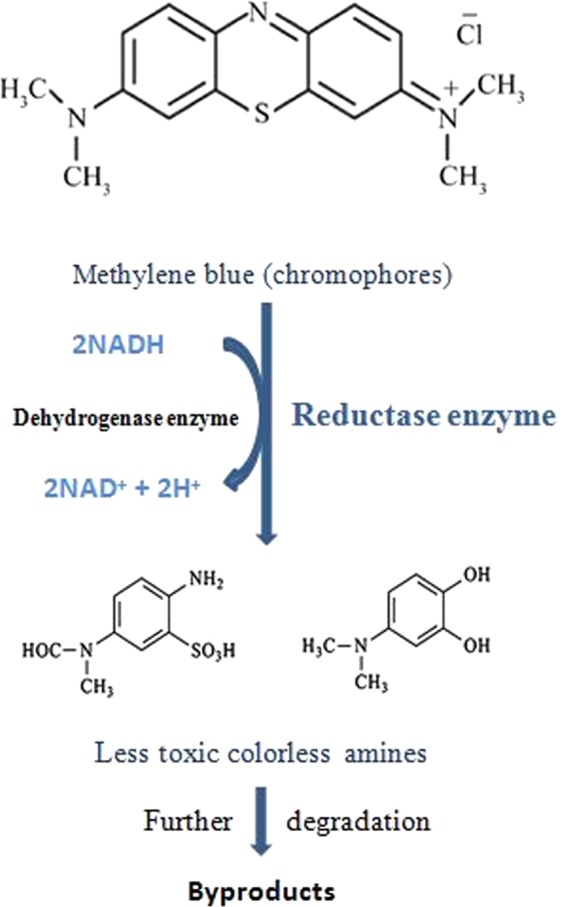
Mechanism of methylene blue biodegradation by bacteria.

Cellulose acetate has been chosen for nanofibrous membrane preparation as it is water-insoluble, biodegradable, biocompatible and highly porous good support for bacteria immobilization^25–28^. As illustrated is Fig. 1, different CA/PEO concentrations (12, 15 and 18 wt%) were electrospun and resulted in various nanofibers morphology. It is seen that 18 wt% CA/PEO concentration produced the highest average nanofibers diameter distribution. Furthermore, the nanofibers diameter distribution of 15 wt% CA/PEO was more uniform and narrower than 12 and 18 wt%. This indicates that 15 wt% CA/PEO produces a less fibers diameter variation, which means that 15 wt% CA/PEO concentration is the optimum polymer concentration for electrospinning process, especially in the presence of DMSO as a new solvent for cellulose acetate- poly(ethylene oxide) electrospinning. In addition, it can be clearly noticed that the bacterial cells are well dispersed and quite a lot adhered into the nanofibers (Fig. 2). Furthermore, this could give us positive expectations for the efficient performance of bacterial cells during methylene blue (MB) decolorization test. Dyes decolorization by bacteria could be attributed to either adsorption on microbial cells or by biodegradation. In case of adsorption, the bacterial cells become colored after the removal process. While in case of biodegradation, the bacterial cells maintain their original color^32^. In this study, no change in the color of bacterial cells was observed in the bacterial cells color after the decolorization process. This observation indicates that the mechanism of decolorization of the isolated bacteria in this study is biodegradation (Fig. 9). Bacteria metabolite organic compounds which contain carbon and nitrogen sources that act as growth substrates in generating reducing equivalents (e.g., reduced nicotinamide adenine dinucleotide; NADH and flavin adenine dinucleotide; FADH_2_). These reducing equivalents are considered the energy source for all biologic oxidative and fermentative systems^33^. Methylene blue has high molecular weight, therefore it cannot penetrate the bacterial cell wall. That is why, the biodegradation reactions occur outside the bacterial cells. The master step in the biodegradation pathway is the cleavage of the double bond to give colorless aromatic amines^34,35^. This reduction occurs by reductases enzymes which are synthesized and released by bacteria into the surrounding media. The presence of oxygen inhibits this reaction and for this it occurs anaerobically. Reductases enzymes utilize NADH and FADH_2_ which are generated by bacterial metabolism as the source of electrons in the reduction step. Dehydrogenases enzymes are further synthesized by bacteria to convert NADH to NAD^+^, these enzymes work antagonistically to reductases in a cycle from successive reactions. Further degradation occurs on the produced amines to convert them to less toxic compounds^3,36^. In Fig. 3, the explanation to the decline in bacterial removal efficiency by the time is the shortage of nutrients in the media with the passage of time after 48 h. As known, biodegradation of methylene blue occurs by reductases enzymes which are produced and secreted by the bacteria into the surrounding media. These enzymes utilize NADH and FADH_2_ in their reactions, which were produced by bacteria metabolism. Therefore, shortage of nutrients could be the cause of NADH and FADH_2_ diminishing. This affects badly on the enzymes efficiency in MB biodegradation^19,20^. The difference in the removal capacity between free bacteria and bacteria-immobilized CA/PEO nanofibrous membrane is very impressive and quite; nevertheless, using bacteria-immobilized CA/PEO nanofibrous membrane has certain advantages than using free bacteria such as good dispersion and easy handling. As the highest MB removal % was obtained after 48 h, the time of 48 h period was selected as the optimum contact time for all coming decolorization tests as the maximum removal occurs. Furthermore, CA/PEO nanofibers exhibited dye adsorption properties and the equilibrium adsorption data was best presented by the Langmuir isotherm model. The calculated free energy is positive value which indicates that the methylene blue adsorption on CA/PEO nanofibers is an endergonic reaction and non-spontaneous in nature (Fig. 7). It can be obviously noticed that the decrease in the decolorization capacity due to increasing in the initial dye concentration, was not significant (Fig. 4). Nevertheless the increase in the initial dye concentration was 10 times from 20 to 200 mg/L, it wasfound that the decrease in the decolorization capacity was only 6% roughly. The results demonstrated in Fig. 5, indicated that when increasing bacteria-immobilized CA/PEO nanofibrous membrane piece area, the MB removal % consequently increased. It is anticipated that the dye removal capacity is relevant to the amount of bacteria and nanofibers mat. Hence, removal efficiency could be improved by increasing both the bacteria and the nanofibers quantity. From reusability results illustrated in Fig. 8, the decline in the removal efficiency after reusability might be due to detachment of some bacterial cells from the nanofibrous membrane at the washing step or death of some cells due to lack of nutrients.

## Materials and Methods

### Materials

The cellulose acetate was purchased from ACROS Organics (USA) (Mw. 100.000 g/mole). Polyethylene oxide was purchased from Sigma Aldrich (Germany) (Mw. 900.000 g/mole). Dimethyl Sulfoxide (DMSO) was purchased from SDFCL, Mumbai (India) (99% (GC)). Methylene blue (MB) dye (extra pure, SLR, C.I.52015, fisher chemical) was purchased from Fisher Scientific (USA). Nutrient agar and nutrient broth were purchased from Sigma Aldrich, Germany.

### Methods

#### Isolation and growth of bacterial cells

Random samples of 50 mL/each were taken from the wastewaters of different factories at Alexandria industrial region, Egypt. Small droplet (50 µL) of each sample was spread on a plate of culture media containing methylene blue dye. Eight bacterial strains were isolated as they were able to degrade methylene blue dye from the culture media. Furthermore, the best strain was selected by testing the MB removal capacity of the isolated strains using known concentration of the methylene blue dye (50 mg/L) and calculating the MB removal % of each of them. The highest MB removal % represented the best bacterial strain (*Bacillus paramycoides*) in methylene blue biodegradation. The methylene blue removal ratio (R %) was calculated from Eq. 4:

4 $${\rm{R}} \% =({{\rm{C}}}_{{\rm{o}}}-{{\rm{C}}}_{{\rm{i}}})/({{\rm{C}}}_{{\rm{o}}})\times 100$$

While C_o_ (mg/L) is the initial concentration before adding bacterial strain and C_i_ (mg/L) is the residual concentration of MB^3^.

#### Bacterial strain identification

The isolated strain was identified by DNA extraction and 16-S-rRNA technique at Sigma Labs, Egypt.

#### Preparation of bacteria-immobilized nanofiberous membrane

Preparation of the electrospinning solution. Cellulose acetate (CA) powder was dissolved in Dimethyl Sulfoxide (DMSO). Polyethylene oxide (PEO) was added with a small ratio (10%) to enhance the spinability of cellulose acetate. The solution was left on the magnetic stirrer until it becomes homogenous, completely dissolved and clear viscous solution. Different concentrations were prepared 12, 15 and 18 wt% of CA/PEO solution.

Immobilization of *Bacillus paramycoides* on CA/PEO nanofibers. The isolated bacterial cells were cultured in a flask containing nutrient broth media and put in a shaker at 30 °C for growth. Nutrient broth (peptone from meat 5.0 g, meat extract 3.0 g and sodium chloride 6.0 g dissolved in 1 L and pH 7). A volume of 80 mL from *Bacillus paramycoides* culture were centrifuged and the cells pellet was taken and put onto the CA/PEO solution before electrospinning.

Electrospinning of CA/PEO solution. In this process, high voltage from 25–30 was applied, the distance between the needle tip to the collector was 15 cm and the feed rate was 1 mL/h. Rotating drum collector was used to collect the nanofibrous mat at a high speed 3000 rpm. After the electrospinning process, the prepared CA/PEO nanofibrous membrane were put into a vacuum oven at 30 °C for 24 h to expel any remained solvent.

#### Characterization methods and techniques

##### Scanning electron microscope (SEM)

Morphology and diameters of the prepared nanofibers were investigated by using (JEOL, JSM-6010LV, Japan) with an accelerating voltage 20 KV. Samples were placed on copper holder and coated with a layer of platinum before scanning.

##### Bacterial cells count on nanofiberous membrane

The bacterial culture was performed in a 250 mL flask and put in a shaker at 30 °C for at least 24 h for growth in order to obtain high optical density (OD)^37^. In our study, the optical density of bacterial culture was obtained by measuring with UV-spectrophotometer at wavelength of 600 nm.

$$({{\rm{OD}}}_{600})=1.27$$

80 mL of the bacterial culture with OD_600_ of 1.27 were taken for centrifugation. The precipitated cells were washed and put onto 4 mL of the CA/PEO blend solution for electrospinning. After the electrospinning process, the fabricated bio-membrane area was measured to calculate the quantity of bacterial cells on each 1 cm^2^. The resulted bio-membrane sample was 3 cm in width and 40 cm in length. The quantity of bacterial cells on each cm^2^ of the immobilized nanofibers was calculated by Eq. 5^37^.

5 $${{\rm{OD}}}_{600}\,{\rm{of}}\,1.0\approx 5\times {10}^{8}{\rm{cells}}/{\rm{mL}}$$

As optical density (OD_600_ = 1.27), so the quantity of free bacterial cells in each mL = 1.27 × 5 × 10^8^ cells. Thus whole bacterial cells count in the prepared electrospun solution equals:

$$1.27\times 5\times {10}^{8}\times 80=508\times {10}^{8}\,{\rm{bacterial}}\,{\rm{cells}}$$

By dividing the whole bacterial cells count in the electrospun solution (508 × 10^8^ bacterial cells) on the area of the fabricated CA/PEO nanofibrous membrane (120 cm^2^), It was concluded that each 1 cm^2^ of bio-membrane contains 4.23 × 10^8^ bacterial cells/cm^2^. The fabricated bio-membrane was cut into pieces with different areas of 1, 2 and 4 cm^2^ to be ready for application.

##### Methylene blue decolorization

Methylene blue solution was prepared by adding methylene blue dye after dissolving in distilled water onto nutrient broth liquid media. A piece of nanofibers 1 cm^2^ was placed into 5 mL MB/liquid media (20 mg/L) and placed into constant temperature shaking incubator at 35 °C for different time intervals as mentioned before. Methylene Blue removal % was measured after each time interval. UV-visible spectrophotometer was used to determine the color absorbance of methylene blue in the supernatant solution before and after application of immobilized nanofibers using a double beam UV-Spectrophotometer (T80 + UV/Vis spectrometer, PG Instruments Ltd, UK) at a 668 nm wavelength^38^. Absorbance of the initial dye concentrations were taken before performing the tests. After the decolorization tests, absorbance of the residual dye concentrations were taken to calculate the MB removal % by the previous equation.

##### Determination of adsorption isotherm

Langmuir isotherm model was used to determine the behavior of methylene blue adsorption on cellulose acetate-poly(ethylene oxide) nanofibers and free energy was calculated to determine the type of reaction.

##### Reusability tests

MB decolorization studies were performed four times to assess the reusability of the bio-membrane for an initial concentration of 20 mg/L. Before each cycle, bio-membrane was washed three times with distilled water. Reusability tests were performed at 35 °C for 48 h.

## Conclusion

This novel bio-membrane has been successfully explored for the biodegradation and adsorption of MB from wastewater. The simultaneous biodegradation and adsorption processes proved to be highly effective for the decolorization of methylene blue dye^39–43^. Approximately 88% of MB removal could be achieved by the bacteria-immobilized CA/PEO nanofibrous membrane after 24 h. Furthermore, after 48 h, around 93% of MB could be removed. Thus, this membrane is quite efficient in MB biodegradation and adsorption which could be considered a very promising membrane. Moreover, it could be used in industry because of its easy handling and effective reusability.
